# Development of pilot scale nanofiltration system for yeast industry wastewater treatment

**DOI:** 10.1186/2052-336X-12-55

**Published:** 2014-03-04

**Authors:** Ahmad Rahimpour, Mohsen Jahanshahi, Majid Peyravi

**Affiliations:** 1 Faculty of Chemical Engineering, Babol University of Technology, Babol, Iran

**Keywords:** Nanofiltration, Membrane process, Pilot scale, Wastewater treatment

## Abstract

The treatment of the yeast industry wastewater was investigated by nanofiltration (NF) membrane process on a pilot scale. Two wastewaters were used as feed: (i) dilute wastewater with COD 2000 mg/L and (ii) concentrate wastewater with COD 8000 mg/L. The permeate flux, COD retention, color and electrical conductivity (EC) removal were evaluated in relation to trans-membrane pressure and long-term filtration. A linear growth in permeate flux was found with increasing in trans-membrane pressure for wastewaters. In addition, the COD retention, color and EC removal increased with trans-membrane pressure enhancement. The results obtained from the long-term nanofiltration of dilute wastewater indicated that the permeate flux decreased from 2300 L/day to 1250 L/day and COD retention increased from 86% to 92%. The quality of the permeate in term of COD is lower than the discharge standard in river (200 mg/L). Thus, this process is useful for treatment of wastewaters produced by yeast industry.

## Introduction

The increasing demand of bread as staple food of human beings has developed backer’s yeast industry. Baker’s yeast industry is an important and developing industry in Iran. In yeast industry, the sugar beet molasses are used as a main raw material. These molasses contains 45–50% residual sugars, 15–20% nonsugar organic substances, 10–15% ash (minerals), and about 20% water [1]. During yeast fermentation, the sugars contained in the molasses are a source of carbon and energy. However, a major part of the non-sugar substances in the molasses are not assimilable by the yeast and are released unchanged to the processing wastewater. These compounds represent the principal waste from the yeast production process [2]. A high chemical oxygen demand (COD), dark color, and high concentrations of total nitrogen (N_tot_) and non-biodegradable organic pollutants are the characteristics of the wastewater produced by yeast industry [3]. Most of the contaminants in the wastewater are due to the use of molasses as a main raw material. Two types of wastewater are produced in Iran's yeast industry; (i) concentrate wastewater with COD of 25000 mg/L; this wastewater is originated from yeast separators and processes such as centrifuges and rotary vacuum filters and (ii) dilute wastewater with COD about 3000 mg/L; this wastewater is created from floor washing and equipment cleaning. The concentrate wastewater is firstly treated by evaporation process. This treated wastewater is combined with dilute wastewater. The COD value of wastewater at this step is about 8000 mg/L. The combined COD is sent to the aerobic treatment stage. The minimum COD at the end of aerobic treatment is about 2000 mg/L. In Iran, each of yeast factories produces about 1000 m3/day of wastewater, which is mostly treated with an anaerobic biological process. The most of yeast factories in Iran has developed and improved their biological wastewater treatment process to reach effluent targets. However, the current technology is still not meeting the environmental requirements as the total treatment efficiency in terms of COD is only about 70–80%.

Successful applications of membrane process for the treatment of industrial wastewater can be found in the recent literature. In fact, membranes technologies provide an important solution in environmental fields such as pollution reduction and water reuse, recycling valuable components from the waste streams [4]. Nanofiltration is a pressure driven membrane process which is somewhere between reverse osmosis and ultrafiltration. The major transport mechanism in nanofiltration membranes is solution-diffusion mechanism. These membranes contain fixed negatively charged functional groups on their surfaces. Both nanofiltration (NF) and reverse osmosis (RO) are good alternatives for wastewater treatment because the high reductions in the conductivity, COD and color can be obtained [5]. Nevertheless, un-treated wastewaters can not be used directly for membrane processes because these have major influence on nanofiltration or reverse osmosis membranes. Therefore, it is necessary to carry out a very exhaustive pre-treatment in order to avoid membrane fouling and membrane deterioration [6,7]. During membrane treatment of yeast wastewater, two flows are generated (i) Filtered stream (permeate) which can also be used as process water in the fermentation industry and (ii) concentrate stream containing high amounts of recalcitrant organics that must be disposed off. Usually, to avoid the discharge of concentrate stream in environment, the concentrate stream is recycled back to the biological treatment plant. Few researches have been carried out about the wastewater treatment of yeast industry, especially with membrane process [8-11]. The most pilot scale nanofiltration processes have been developed for treatment of textile industry wastewater [12-15]. The main objective of this work was to investigate the application of pilot plant of nanofiltration process for treatment of the yeast industry wastewater. The ability of this pilot plan to COD retention, color and conductivity removal of wastewater was investigated.

## Experimental

### Wastewater

Two different wastewaters were used as feed for nanofiltration plan. One is the full biological treated wastewater with COD about 2000 mg/L and the other one is the evaporational treated wastewater with COD about 8000 mg/L. These wastewaters were named as dilute and concentrate wastewaters, respectively. The detail characteristics of these wastewaters were given in Table 1.

**Table 1:** Characteristics of dilute and concentrate wastewaters

**Parameter**	**Dilute wastewater**	**Concentrate water**
pH	6.5	6.0
COD, mg/L	2000 ± 100	8000 ± 220
SS, mg/L	43 ± 5	180 ± 15
Color, Pt-Co^†^	6400 ± 140	14000 ± 660
Conductivity, mS/cm	3200 ± 110	9880 ± 400

† Platinum - Cobalt.

### Equipment

#### Nanofiltration membrane module

The NE4040-90 nanofiltration membrane module was used in pilot scale setup. This nanofiltration membrane module was purchased from CSM, Korea. The membrane module is in a spiral wound form and has a filtration area of 7.9 m^2^. This thin-film composite membrane was made from interfacial polymerization of trimesoyl chloride and *m*-phenylene diamine (TMC/mPDA) on polysulfone micro-porous membrane with a non-woven polyester backing. The rejection ranges for sodium chloride and magnesium sulfate are 85–95% and 99.5%, respectively (reported by manufacture). The maximum free chloride concentration is 0.1 mg/L. The maximum operating pressure and temperature are 40 bar and 45°C, respectively. The permitted operating pH range is 2–11. In addition, the maximum feed flow rate and minimum concentrate flow rate are 4 and 0.91 m^3^/hr, respectively.

#### Ultrafiltration membrane cartridge

The ultrafiltration membrane cartridge (PUF-6040) used for the main pre-treatment of wastewater was provided from Hangzhou Tianchuang Water pure Equipment Co., China. It was a hollow fiber membrane with the high strength, high flux and best anti-pollution. The small-suspended solids were removed by ultrafiltration. PUF-6040 has an effective membrane filtration area of 45 m^2^. The molecular weight cut-off of this membrane is about 80000 Da. The pure water permeate flux is 100 (1/m^2^.h.atm) at 25°C. Maximum operating pressure and temperature are 4 bar and 50°C, respectively. The detailed characteristics of ultrafiltration cartridge were listed in Table 2.

**Table 2:** Characteristics of hollow fiber ultrafiltration cartridge

**Parameter**	**Value**
Membrane type	Out-to-In hollow fiber
Membrane material	PP(Polypropylene)
Housing	UPVC/ABS
Pore size	0.1~0.2 μm
Fiber OD/IN	OD450 μm,ID3500 μm
Ventilation rate of N_2_	≥7.0 × 10–2 cm^3^/cm^2^.cmHg
Porosity rate	40~50%
Strength	12 MPa
Product water turbidity	≤ 0.2 NTU
Product water	SDI ≤ 3
TOC removing rate	20% ~ 50%

### Pre-treatment

Due to the presence of suspended solids in the yeast industry wastewater, pre-treatment of the wastewater is necessary to prevent the NF membrane fouling, plugging and deterioration. This may lead to decline in flux and rejection or even membrane failure. Two stages were selected for initial pre-treatment of wastewaters; sand filter and cartridge depth micro-filter. The vessel of sand filter was filled with three type of silica; large, medium and fine particles, respectively. The cartridge depth filter is a polypropylene porous membrane with a pore size of 10 micron. After these two steps, the majority of large and medium particles were removed from the wastewaters. This is very important to prevent the ultrafiltration and nanofiltration membrane from damage.

### Pilot scale nanofiltration setup

**Figure 1:**
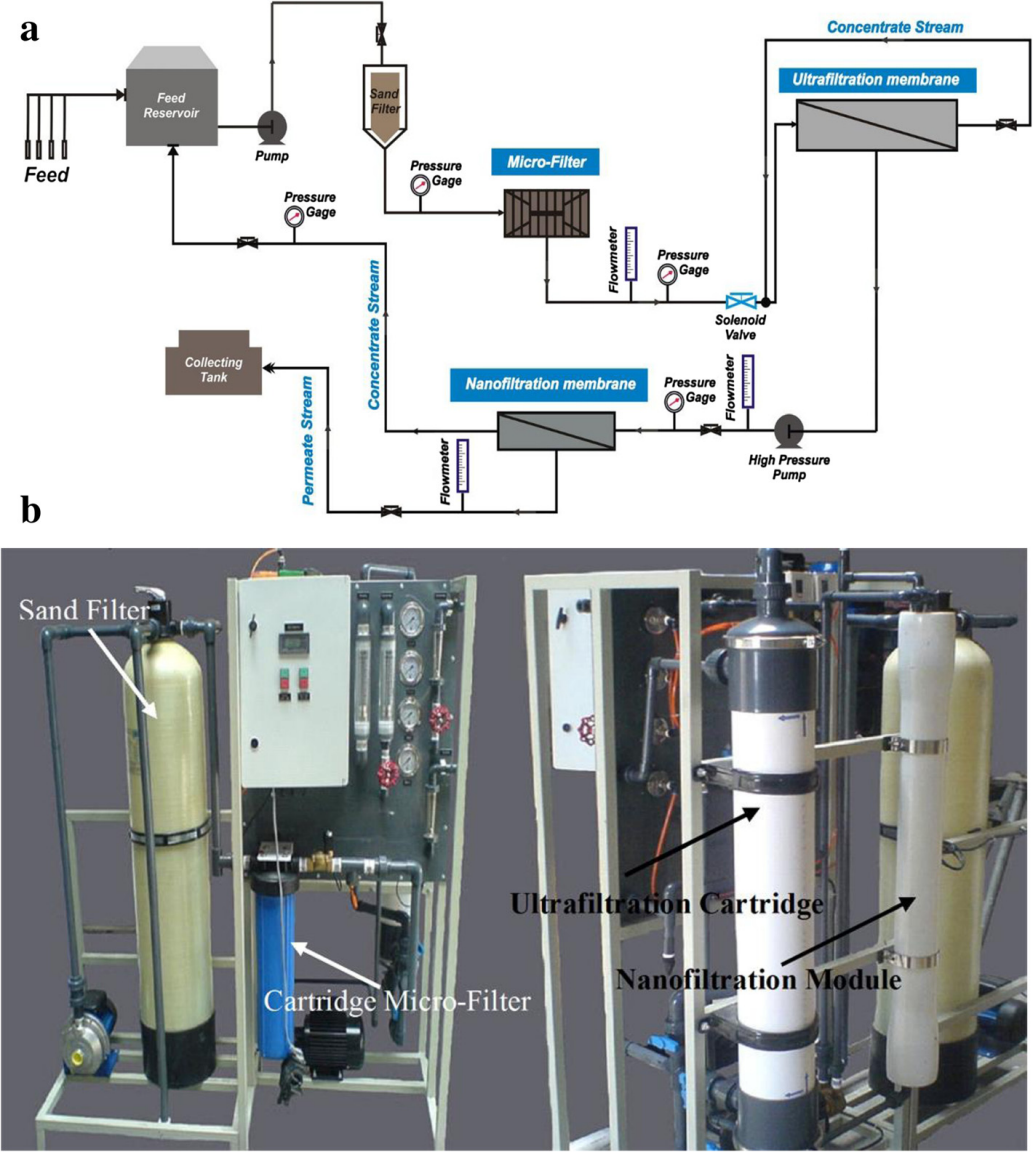
Nanofiltration system for yeast wastewater treatment: (a) flow diagram (b) image.

The flow diagram and images of designed nanofiltration system for treatment of yeast wastewater were shown in Figure 1. As shown, immediately after turning on the system, the centrifugal pump started to operate and the wastewater passed through the sand and cartridge depth filters as initial pre-treatment equipments. The large and medium particles that could cause to damage of the ultrafiltration membrane were removed at the end of these two stages. This stream was entered to ultrafiltration cartridge. The concentrate stream of ultrafiltration module was re-circulated to this module and the permeate stream was passed through the nanofiltration membrane module by high-pressure pump. The concentrated stream of nanofiltration module was recycled to the feed tank. Also, the permeate stream of nanofiltration module was collected in the separate tank. The permeate and concentrate flow rates for ultrafiltration and nanofiltration module were measured by two rotameters, separately. For indicating the inlet and outlet pressure of each step, four oily pressure gauges were used. The backwash of ultrafiltration cartridge was carried out by using four valves set in the system. The performance of NF membrane was measured during 1 hr of filtration. The electrical conductivity and color of the solutions were determined by using digital conductivity meter (AZ86505) and spectrophotometer (ColorFlex EZ Citrus), respectively.

## Results and discussions

### Permeate flux of nanofiltration system during filtration of wastewaters

**Figure 2:**
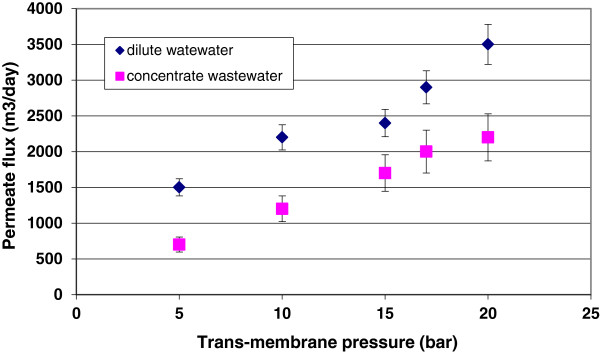
Effect of trans-membrane pressure on permeate flux of pilot plan.

The passage of water through the membranes or permeability, which is an important parameter in the design and economical feasibility analysis of membrane separation processes, depends on several parameters including trans-membrane pressure, operating temperature, feed velocity, feed solution concentration and membrane properties such as porosity, material, hydrophilicity, roughness and charge. The relationship between permeate flux and trans-membrane pressure of nanofiltration system during treatment of dilute and concentrate wastewaters was shown in Figure 2. The nanofiltration process showed an acceptable permeate flux at pressures ranging from 15 to 20 bar particularly during treatment of dilute wastewater.

As shown in this figure, the permeate flux of nanofiltration membrane increased proportionally with increasing of the trans-membrane pressure. This confirmed that the operation was in the pressure-controlled region. As expected, flux increased linearly with increasing of trans-membrane pressure. Spiegler-Kedem Model [16] can describe this behavior:

(1) $$J_{v}=L_{p}\left(\mathrm{\Delta P}-\sigma \Delta \pi \right)$$

where *J*_v_ is the water flux, *L*_p_ is the pure water permeability, Δ*P* is the trans-membrane pressure (TMP), *σ* is the reflection factor of the membrane, and Δ*π* is the osmosis pressure. This equation shows the permeate flux (J_v_) has linear relationship with trans-membrane pressure (Δ*P*).

The significant difference in permeate flux between two kinds of wastewater was observed. The permeate flux of nanofiltration system during filtration of dilute wastewater was higher than concentrate wastewater. The decline in permeate flux was nearly 51% for a change in the wastewater concentration from COD of 2000 mg/L to COD of 8000 mg/L. It is expected that the permeate flux would decrease for the concentrate wastewater rather than the dilute wastewater due to concentration polarization. Concentration polarization was obtained from the gradual increase of the boundary layer of highly concentrated solute on the membrane surface. This term is used to describe the accumulation of membrane-rejected solutes close to the membrane surface. As water passes through the membrane, the convective flow of solute to membrane surface is much higher than the diffusion of the solute back to the bulk feed solution. Consequently, the concentration polarization reduces the permeate flux of the membranes.

### COD retention, color and EC removal

**Figure 3:**
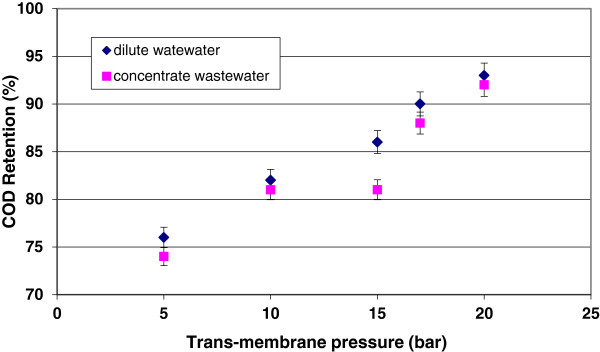
Effect of trans-membrane pressure on COD retention of pilot plan.

Figure 3 shows the relationship between COD retention and trans-membrane pressure of the nanofitration system during dilute and concentrate wastewater treatment. The range of COD retention was 76 to 93% for dilute waste water and 78 to 92% for concentrate wastewater when trans-membrane pressure increased from 5 bar to 20 bar. At the best condition, the COD of dilute and concentrate wastewaters were reduced to 140 and 640 mg/L. Therefore, the performance of this pilot plan in term of COD reduction can be considered satisfactory.

**Figure 4:**
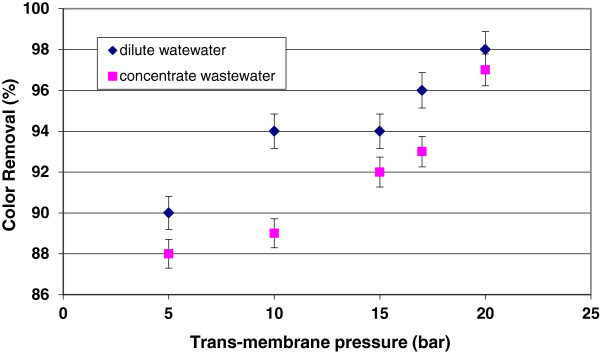
The relationship between color removal and trans-membrane pressure.

**Figure 5:**
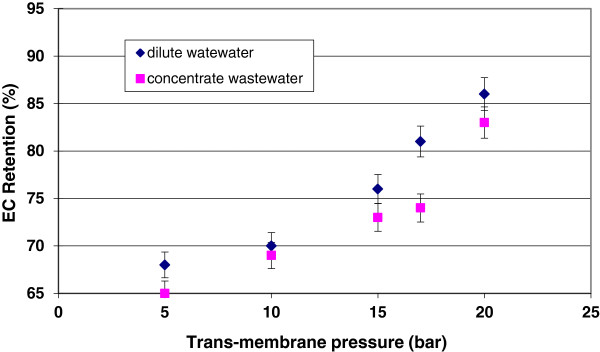
Effect of trans-membrane pressure on EC retention of pilot plan.

The increase in COD retention when pressure increases, is due to the level of concentration polarization and cake layer formation that induce a higher rejection rate in the pressure controlled region. Generally, the deposition of particles/macromolecules on the surface of the membrane leads to the formation of a “gel” layer, was usually denominated “secondary membrane”. When the pressure above the gel layer increases, the compaction of this layer increases. This phenomenon results in to pass the fewer amounts of ions and organic compounds through the membrane. Therefore, an increment in levels of COD retention is observed. However, the flux and cake enhanced osmotic pressure (CEOP) effect of fouling in nanofiltration were raised. This phenomenon can be considered as a negative effect and must be controlled by operating conditions and cleaning.Figures 4 and 5 indicate the effect of trans-membrane pressure on color removal and EC retention during filtration of dilute and concentrate wastewaters. When pressure increased from 5 to 20 bar, the color removal increased from 90 to 98% for dilute wastewater and 88 to 97% for concentrate wastewater. EC retention of nanofiltration system increased from 68 to 86% for dilute wastewater and 65 to 83% for concentrate wastewater. The higher retention for color and EC at higher pressure can be explained by the higher compaction of cake layer formed on the membrane surface at higher pressures. In fact, the electrical conductivity has a direct relationship with ionic diffusivities through the membrane surface. the deposition of particles/macromolecules on the membrane surface is occupied the pores together with electro-migration occurring at the cake layer–surface interface in the electrical double layer.

### Long-term performance of pilot plan

**Figure 6:**
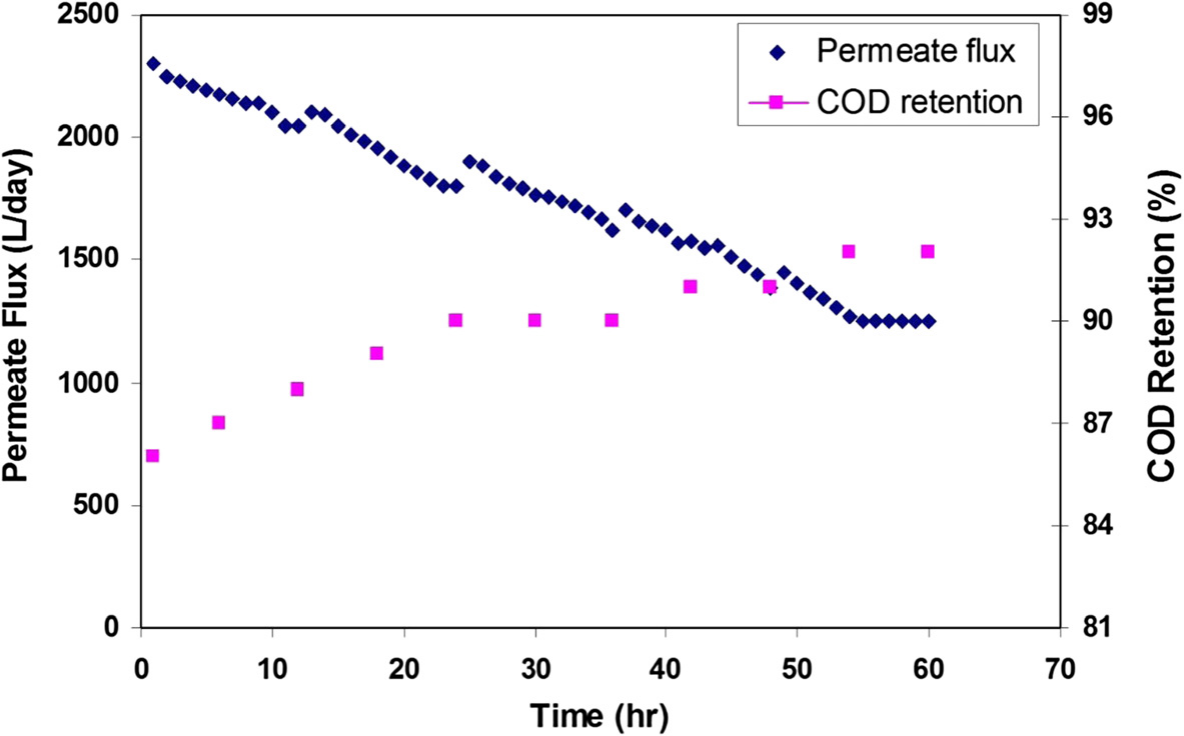
The permeate flux and COD retention of pilot plan during long-term filtration.

Since the initial performance of pilot scale was very successful and promising, it was decided to carry out the long term experiment. Long-term experiment provides reliable information about fouling rates and long-term stability of the membrane. During a period of 5 days, the pilot nanofiltration plan has been tested in a continuous mode at 15 bar and 25°C. In each day, the deigned system operated for 12 hr and then washed with clean water about 1 hr. During this experiment, the nanofiltration performance has been determined by measuring permeate flux and COD retention as a function of time. Figure 6 indicates the changes in the permeate flux and COD retention of the pilot scale nanofiltration system during treatment of the dilute wastewater for 60 hr at 5 runs. For each run, the flux decreased gradually. This is a typical behavior of membrane processes. The flux is controlled by two phenomena: concentration polarization and membrane fouling. As mentioned earlier, the concentration polarization results from the gradual increase of the boundary layer of highly concentrated solute on the membrane surface. As a result, water flux of membrane decreases. The concentration polarization is removed by washing the system. Thus, it can be concluded that the membrane fouling is the major factor to decline the water flux. Fouling is one of the major problems in any membrane separation process, but for nanofiltration it might be even somewhat more complex because of the interactions leading to fouling take place at nanoscale, and are difficult to understand [17]. This phenomenon is a process resulting in loss of performance of a membrane due to the deposition of suspended or dissolved substances on external surface, at its pore openings or within its pores. The Majority of foulants in nanofiltration membrane process can be organic solutes, inorganic solutes, colloids, or biological solids [18]. The membrane fouling is closely related to other characteristics of membrane process such as concentrate treatment and membrane stability and lifetime. Therefore, a total control of fouling would reduce the need for cleaning and would enhance the permeate yield. For aqueous applications, the membrane lifetime depends significantly on the cleaning frequency and the overall strategy against the membrane fouling.As seen in Figure 6, the COD retention of pilot scale nanofiltration system increased from 86% to 92% after 60 hr filtration of wastewater. This increase in COD retention can be explained by cake formation onto the surface of nanofiltration membrane due to fouling. Thus, the COD of dilute wastewater decreased from 2000 mg/L to 160 mg/L. The standard and allowable COD for wastewater to discharge in river is 200 mg/L. Therefore, the final COD of treated dilute wastewater is much lower than the standard for Iran river discharge. The significant reduction in COD, color and conductivity makes possible the treatment of yeast industry wastewater by designed pilot scale nanofiltration system.

## Conclusion

The current study shows that the pilot scale nanofiltration process is technical and efficient process for treatment of the yeast industry wastewater. The permeate flux, COD retention, color and EC removal enhanced with increasing trans-membrane pressure during filtration of dilute and concentrate wastewaters. The long-term performance of pilot scale setup was investigated by filtration of dilute wastewater. The obtained results showed that the permeate flux decreased from 2300 L/day to 1250 L/day and the COD retention increased from 86% to 92%. It denoted that the COD of dilute wastewater decreased from 2000 mg/L to 160 mg/L. Therefore, the quality of the permeate in term of COD was lower than the discharge standard in river (200 mg/L).

## Competing interests

All authors declare that they have no competing interest.

## Authors’ contributions

AR participated in the design of the study and supervised the work. AR, MJ and MP did the analyses, and/or interpreted the analyzed results. AR and MP wrote the initial draft and revised the paper critically for important intellectual content and compiled the work in accordance to journal format. All authors have read and approved the final manuscript.
